# Supplementary material for: Regulation of zebrafish melanocyte development by ligand-dependent BMP signaling
Source: eLife. 2019 Dec 23;8:e50047. doi: 10.7554/eLife.50047 (PMC6968919; doi:10.7554/eLife.50047)
Supplement: Supplementary file 1. — (A) Primers used for qRT-PCR reactions. (B) Primers used for genotyping PCR reactions and generation of Gateway compatible DNA fragments. [file elife-50047-supp1.docx]

**Supplementary File 1**

| **A. qPCR Primers** |  |
| --- | --- |
| Beta-actin Fwd | GGTTTTGCTGGAGATGATGC |
| Beta-actin Rev | GGGGTATTTGAGGGTCAGGA |
| Gdf6b Fwd | CGTCTAAAGCAGCAAACACC |
| Gdf6b Rev | CCAAAGTGGAGAGTTCAAATGG |
| Mitfa Fwd | CTGGACCATGTGGCAAGTTT |
| Mitfa Rev | GAGGTTGTGGTTGTCCTTCT |
| Tyrp1b Fwd | CGACAACCTGGGATACACCT |
| Tyrp1b Rev | AACCAGCACCACTGCAACTA |
| Neurog1 Fwd | GTCGTGAAGAAGAACCGCAG |
| Neurog1 Rev | AGCTGTACACTACGTCGGTT |
| Pnp4a Fwd | GAAAAGTTTGGTCCACGATTTC |
| Pnp4a Rev | TACTCATTCCAACTGCATCCAC |
| Mpba Fwd | GAGGAGACAAGAAGAGAAAGGG |
| Mbpa Rev | GAAATGCACGACAGGGTTG |
| Pomca Fwd | TTTCTGTGCAAAAGAGATGGTG |
| Pomca Rev | ATAATTGTATGCATTCCAAGATGTTC |
| Aox5 Fwd | AGGGCATTGGAGAACCCCCAGT |
| Aox5 Rev | ACACGTTGATGGCCCACGGT |
| Col2a1a Fwd | CCTCTGAAATCCAGCCATGT |
| Col2a1a Rev | GACTGCTGTGGTTCCAGTCA |
| Sox10 Fwd | GGCTGCAGGGTCACCATT |
| Sox10 Rev | AGGGCTGTGACTCTGACCTGTAG |
| Foxd3 Fwd | CCAAGAGCAGCCTGGTAAAG |
| Foxd3 Rev | CAGATTCCACTGAGCGTCAA |
| Tyr Fwd | GGATACTTCATGGTGCCCTT |
| Tyr Rev | TCAGGAACTCCTGCACAAAC |
| Mc1r Fwd | TTCTTCCTCCACCTCATCCT |
| Mc1r Rev | CTGACTGCGGTAAGCGTAAA |
| Gdf6a Fwd | GAGCTTTTTCCGCTCTTCAA |
| Gdf6a Rev | CCTTAATTCAGCACCGACCA |
|  |  |
| **B. PCR Primers** |  |
| Gdf6a genotyping Fwd | ATGGATGCCTTGAGAGCAGTC |
| Gdf6a genotyping Rev | TTGAAGAGCGGAAAAAGCTC |
| Gdf6b genotyping Fwd | AAGATGTGTGCCTGCGGGATAC |
| Gdf6b genotyping Rev | GCTTTAGACGAGCGGAAAAAACTC |
| dnBMPR (attB) Fwd | GGGGACAAGTTTGTACAAAAAAGCAGGCTGAACCATGAAATCCAATTCAGACC |
| dnBMPR (attB) Rev | GGGGACCACTTTGTACAAGAAAGCTGGGTTTACTCATTTCTAGACTG |
